# The Title of Physician

**Published:** 1898-06-25

**Authors:** 


### THE TITLE OF PHYSICIAN.

Correspondence concerning the diploma of the Apothecaries' Society is getting rather heated, and the great question whether an L.S.A. may properly call himself a physician has excited an L.R.C.P.Edin. to remark that "an intolerable amount of snobbishness" is going on anent the designation "Physician and Surgeon." But, after all, what is the poor L.S.A. to call himself? Nay, we may go further, and ask what is the L.R.C.P. to call himself? They have each gone through the same curriculum; they have each been examined by a legally appointed body; they are each exactly on an equality before the law; and they have each obtained a licence to practise medicine, surgery, and midwifery. They are neither of them, however members of the body by which their licence is granted, and the mere fact that this is in one case given by a College of Physicians, and in the other by a Society of Apothecaries is a matter of small moment. It is specifically stated in the bye-laws of the London College that the licences "do not extend to make the Licentiates members of the Corporation" any more, in fact, than the licence of the Apothecaries' Society makes a man a member of that ancient body. If both L.R.C.P. and L.S.A. will describe themselves by their proper titles as Licentiates, &c., they will be right, as also they will if they call themselves legally qualified medical practitioners. This they both are. But this quarrelling about the title of physician is but a matter of the pot calling the kettle black.

